# Ethics of DNA Research on Human Remains: Five Globally Applicable Guidelines

**DOI:** 10.1038/s41586-021-04008-x

**Published:** 2021-10-20

**Authors:** Songül Alpaslan-Roodenberg, David Anthony, Hiba Babiker, Eszter Bánffy, Thomas Booth, Patricia Capone, Arati Deshpande-Mukherjee, Stefanie Eisenmann, Lars Fehren-Schmitz, Michael Frachetti, Ricardo Fujita, Catherine J. Frieman, Qiaomei Fu, Victoria Gibbon, Wolfgang Haak, Mateja Hajdinjak, Kerstin P. Hofmann, Brian Holguin, Takeshi Inomata, Hideaki Kanzawa-Kiriyama, William Keegan, Janet Kelso, Johannes Krause, Ganesan Kumaresan, Chapurukha Kusimba, Sibel Kusimba, Carles Lalueza-Fox, Bastien Llamas, Scott MacEachern, Swapan Mallick, Hirofumi Matsumura, Ana Y. Morales-Arce, Giedre Motuzaite Matuzeviciute, Veena Mushrif-Tripathy, Nathan Nakatsuka, Rodrigo Nores, Christine Ogola, Mercedes Okumura, Nick Patterson, Ron Pinhasi, Samayamantri P.R. Prasad, Mary E. Prendergast, Jose Luis Punzo, David Reich, Rikai Sawafuji, Elizabeth Sawchuk, Stephan Schiffels, Jakob Sedig, Svetlana Shnaider, Kendra Sirak, Pontus Skoglund, Viviane Slon, Meradeth Snow, Marie Soressi, Matthew Spriggs, Philipp W. Stockhammer, Anna Szécsényi-Nagy, Kumarasamy Thangaraj, Vera Tiesler, Ray Tobler, Chuan-Chao Wang, Christina Warinner, Surangi Yasawardene, Muhammad Zahir

**Affiliations:** 1 Department of Genetics, Harvard Medical School, Boston, MA, USA; 2 Department of Evolutionary Anthropology, University of Vienna, Austria; 3 Department of Anthropology, Hartwick College, Oneonta, NY, USA; 4 Department of Human Evolutionary Biology, Harvard University, Cambridge, MA, USA; 5 Max Planck Institute for the Science of Human History, Jena, Germany; 6 Romano-Germanic Commission of the German Archaeological Institute, Frankfurt am Main, Germany; 7 Francis Crick Institute, London, UK; 8 Peabody Museum of Archaeology and Ethnology, Harvard University, MA, USA; 9 Department of Ancient Indian History Culture and Archaeology, Deccan College Post Graduate and Research Institute, Pune, India; 10 Max Planck Institute for Evolutionary Anthropology, Leipzig, Germany; 11 Department of Anthropology, University of California, Santa Cruz, CA, USA; 12 UCSC Genomics Institute, University of California, Santa Cruz, CA, USA; 13 Department of Anthropology, Washington University in St. Louis, St. Louis, MO, USA; 14 Centro de Genética y Biología Molecular, Facultad de Medicina, Universidad de San Martín de Porres, Lima, Peru; 15 School of Archaeology and Anthropology, The Australian National University, Canberra, Australia; 16 Key Laboratory of Vertebrate Evolution and Human Origins, Institute of Vertebrate Paleontology and Paleoanthropology, Center for Excellence in Life and Paleoenvironment, Chinese Academy of Sciences, Beijing, China; 17 Division of Clinical Anatomy and Biological Anthropology, Department of Human Biology, University of Cape Town, Cape Town, South Africa; 18 Department of Anthropology, University of California, Santa Barbara, CA, USA; 19 School of Anthropology, University of Arizona, Tucson, AZ, USA; 20 National Museum of Nature and Science, Ibaraki, Japan; 21 Florida Museum of Natural History, Gainesville, FL, USA; 22 Department. of Genetics, School of Biological Sciences, Madurai Kamaraj University, Tamil Nadu, India; 23 Department of Anthropology, University of South Florida, Tampa, FL, USA; 24 Institute of Evolutionary Biology (CSIC-UPF), Barcelona, Spain; 25 Australian Centre for Ancient DNA, School of Biological Sciences and The Environment Institute, University of Adelaide, Adelaide, SA, Australia; 26 ARC Centre of Excellence for Australian Biodiversity and Heritage, University of Adelaide, Adelaide, SA 5005, Australia; 27 Office of the Chancellors, Duke Kunshan University, Jiangsu, China; 28 Howard Hughes Medical Institute, Boston, MA, USA; 29 Broad Institute of MIT and Harvard, Cambridge, MA, USA; 30 School of Health Science, Sapporo Medical University, Sapporo, Hokkaido, Japan; 31 Institute of Ecology and Evolution, University of Bern, Bern, Switzerland; 32 Lithuanian Institute of History and Department of Archaeology, History Faculty, Vilnius University, Vilnius, Lithuania; 33 Departamento de Antropología, Facultad de Filosofía y Humanidades, Universidad Nacional de Córdoba, Instituto de Antropología de Córdoba (IDACOR), CONICET, Córdoba, Argentina; 34 Earth Sciences Department, National Museums of Kenya, Nairobi, Kenya; 35 Department of Genetics and Evolutionary Biology, University of São Paulo, São Paulo, Brazil; 36 DBT-Centre for DNA Fingerprinting and Diagnostics, Hyderabad, India; 37 Department of Anthropology, Rice University, Houston, TX, USA; 38 Instituto Nacional de Antropología e Historia, Michoacán, Mexico; 39 School of Advanced Sciences, The Graduate University for Advanced Studies (SOKENDAI), Hayama, Kanagawa, Japan; 40 Department of Anthropology, University of Alberta, Edmonton, Alberta, Canada; 41 Department of Anthropology, Stony Brook University, Stony Brook, NY, USA; 42 ArchaeoZOOlogy in Siberia and Central Asia – ZooSCAn, CNRS – IAET SB RAS International Research Laboratory, Novosibirsk, Russia; 43 Department of Anatomy and Anthropology and Department of Human Molecular Genetics and Biochemistry, Sackler Faculty of Medicine, Tel Aviv University, Tel Aviv, Israel; 44 The Dan David Center for Human Evolution and Biohistory Research, Tel Aviv University, Tel Aviv, Israel; 45 Anthropology Department, University of Montana, Missoula, MO, USA; 46 Faculty of Archaeology, Leiden University, Leiden, Netherlands; 47 Vanuatu Cultural Centre, Port Vila, Vanuatu; 48 Institute for Pre- and Protohistoric Archaeology and Archaeology of the Roman Provinces, Ludwig Maximilian University, Munich, Germany; 49 Institute of Archaeogenomics, Research Centre for the Humanities, Eötvös Loránd Research Network, Budapest, Hungary; 50 CSIR-Centre for Cellular and Molecular Biology, Hyderabad, India; 51 School of Anthropological Sciences, Universidad Autónoma de Yucatán, Mérida, Mexico; 52 Department of Anthropology and Ethnology, Institute of Anthropology, School of Sociology and Anthropology, and State Key Laboratory of Cellular Stress Biology, School of Life Sciences, Xiamen University, Xiamen, China; 53 School of Basic Medical Sciences, Zhejiang University School of Medicine, and Institute of Asian Civilizations, Zhejiang University, Hangzhou, China; 54 Department of Anthropology, Harvard University, Cambridge, MA, USA; 55 Department of Anatomy, University of Sri Jayewardenepura, Nugegoda, Sri Lanka; 56 Department of Archaeology, Hazara University, Mansehra, Pakistan

## Abstract

We are a group of archaeologists, anthropologists, curators, and geneticists representing 24 countries and diverse global communities, most of whom met in November 2020 in a virtual workshop dedicated to ethics in ancient DNA research. There was widespread agreement that globally applicable ethical guidelines are needed, but that recent recommendations grounded in discussion about research on human remains from North America are not always generalizable worldwide. Taking into consideration diverse contexts, we propose the following globally applicable guidelines. These hold that: 1) researchers must ensure that all regulations were followed in the places where they work and from which the human remains derived; 2) researchers must prepare a detailed plan prior to beginning any study; 3) researchers must minimize damage to human remains; 4) researchers must ensure that data are made available following publication to allow critical reexamination of scientific findings; and 5) researchers must engage with other stakeholders from the beginning of a study and ensure respect and sensitivity to stakeholder perspectives. We commit to adhering to these guidelines and expect they will promote a high ethical standard going forward.

## Introduction

The analysis of ancient human genomes has emerged as a powerful approach for investigating the relationships of people who lived in the past to each other and to people living today. A consistent theme is that people in any given location across time are usually there as the result of a long history of mobility and interaction. Over the last decade, ancient DNA has provided new evidence—adding to that from other disciplines—refuting myths of the ‘purity’ of any population and falsifying racist and nationalistic narratives. While some have sought to misuse genetics as a tool for determining group belonging, in our opinion it is inappropriate for genetic data to be used as an arbiter of identity^1^.

The rapid increase in published genome-wide data from ancient humans—from none in 2009 to more than five thousand individuals today—has been accompanied by growing discussions about how to conduct ancient DNA research ethically^2–16^, building on earlier conversations^17–24^. The ethics of DNA research has a particular urgency because of the rapid growth of the field, the social and political impacts of studying ancestry, and the fact that ancient DNA work analyzes once-living people who must be respected.

Institutional or governmental guidelines for obtaining permission to analyze ancient individuals vary and do not always ensure ethical and engaged research. Researchers have an obligation to meet a higher standard than some governing bodies may require, but there is no consensus on what this entails^11,25–27^. Increasingly, ancient DNA publications have included statements describing how the research team addressed ethical issues^28–38^, a development we support. Professional organizations are also beginning to articulate guidelines^15,39^, and at least one grant has been awarded to explore these issues in the context of research on ancient North Americans^40^. Notably lacking has been a statement on ethics co-signed by an internationally diverse and representative group of scholars engaged in ancient DNA research.

We convened more than sixty archaeologists, anthropologists, curators, and geneticists representing over twenty countries and diverse global communities for a virtual workshop on ethics from November 4-5, 2020. All participants are committed to carrying out research on DNA from human remains that is ethically responsible and sensitive to diverse perspectives held by stakeholders (people who have a connection to a study, including descendant communities, those responsible for the stewardship of human remains, and researchers). Here we present case studies from a variety of global contexts to illustrate the breadth of issues surrounding community and Indigenous group consultation, highlighting how the relevant issues vary worldwide. We then provide guidelines for DNA research on human remains that apply globally (Box 1).

## Ethical engagement with communities is context-specific

Much of the literature about ethical DNA research on ancient individuals has focused on the United States (US)^3,4,13,15^. These discussions have produced recommendations to promote engagement between researchers and Indigenous communities, summarized in a Research Guidance published by the American Society of Human Genetics suggesting that all ancient DNA studies should involve formal consultation, address cultural and ethical considerations, engage communities and support capacity-building, develop plans for reporting and managing data, and develop plans for long-term responsibility and stewardship^15^.

Centering Indigenous perspectives is critical in regions with histories of settler colonialism, expropriation of Indigenous lands and artifacts, and persistent disenfranchisement of Indigenous communities; not consulting with communities can cause harm in such contexts^5,6^. In the US, all ancient Native American remains held in federally funded institutions fall under the purview of the Native American Graves Protection and Repatriation Act (NAGPRA), which mandates that institutions consult with and seek to transfer the remains of ancient individuals (culturally identifiable or not) to Indigenous groups. In Australia, analogous laws seek to repatriate human remains in some cases up to 40,000 years old^41^ removed from Aboriginal and Torres Strait Islander communities^42,43^. However, when carrying out research on the remains of ancient individuals where there are few (if any) material or oral links to present-day groups, or where promoting the idea that some groups have more ownership of cultural heritage than others can contribute to social conflict, an Indigenous-centered ethical framework mandating that each ancient individual be associated with a contemporary group does not fit.

### Government institutions are sometimes an effective channel to represent Indigenous perspectives

In many countries in the Americas, Indigenous heritage is embedded in national identity and integrated into governmental cultural institutions. For instance, following Independence in Mexico, mestizos (people of mixed ancestry) who form the great majority, embraced legacies from the Nahua (Aztec), Maya, Zapotec, and other Indigenous groups as an integral part of national identity^44–46^. In Peru, the Ministry of Culture was created in the context of *indigenismo*, a movement with the goal of promoting Indigenous culture and fighting discrimination^47–49^. In such contexts, the process of seeking approval from government or heritage organizations for analysis of human remains can be a robust form of engagement, and adopting a US template can be counterproductive. Authors of this article have had multiple experiences of writing papers about ancient DNA from Central and South America and receiving reviews stating the work did not conform to standards for Indigenous engagement developed in the US^3,4^. Those of us who are from Mexico and Central and South America have felt that such reviews have been paternalistic at best and colonialist at worst, especially given that many places have embraced Indigenous heritage and embedded it into government approval processes and cultural institutions to a greater extent than has been done in the US.

There is great variation, however, in the nature of relationships between governments and Indigenous communities in the Americas, and researchers must take a case-by-case approach to determining when additional consultation is needed. In Peru and Mexico, groups for whom Indigenous heritage is an important part of identity have variable degrees of representation in the government. In Brazil, Indigenous communities are often disenfranchised, and there is no legal mechanism for Indigenous groups to have a voice in the fate of archaeological materials associated with their ancestors^50^. In Argentina, a legal mandate that community consent must be obtained to carry out any project involving Indigenous heritage is not always followed. In Guatemala, the Maya and other Indigenous groups who form roughly half the population remain marginalized. In such contexts it is the ethical responsibility of members of an ancient DNA research team to carry out additional outreach beyond what is mandated to incorporate Indigenous perspectives.

### Global differences in the meaning of Indigeneity

The meaning of Indigeneity varies globally. In Africa, descendants of colonized groups are now overwhelmingly in power, and Indigeneity often refers more to political or social marginalization on the basis of identity than to traditions of how long groups have been established in a region^51^. Many African communities have complex connections to the lands on which they live, including histories of colonial and postcolonial displacement and disruption. In some regions, people do not recognize past local populations as their relatives. This may be due to contemporary religious or cultural belief systems being different from past ones^52^, collective memories of migrations from elsewhere, fear of reprisal for being linked with other groups, and the continuing aftershocks of decisions made during European colonization that fractured socio-political landscapes and still contribute to violence and displacements. In these situations, careful consultation among stakeholders is necessary, from local groups to government representatives, to ensure that vesting decision-making power about cultural heritage does not aggravate social conflict. In such cases, centering Indigeneity as a principle for permitting ancient DNA analysis would likely be harmful.

A more pressing issue related to ancient DNA research in Africa (and in many other regions) is confronting the colonial legacies of human remains collected in unethical ways and often sent abroad^53,54^. Researchers must work with both the curating institution and with scholars from the country of origin to seek permissions to study the remains of ancient individuals, and engage in discussions about provenance, historical injustices, repatriation, and restitution as part of their work^55–58^. A related challenge is the history of non-equitable and often exploitative research in Africa by predominantly European and North American scientists, with minimal local engagement^25–27^. Foreign researchers must prioritize establishing equitable collaborations, which may include training and other capacity-building that empowers stakeholders to shape research questions and designs^59^.

### Emphasizing group identity has the potential to cause harm in some global contexts

There are many places in the world where discussions about who is Indigenous have contributed to xenophobic and nationalistic narratives. In these places, using Indigenous identity to determine who can permit ancient DNA research can be harmful, as it can contribute to conflict among groups and to discrimination.

In India, for example, many people avoid asking about caste and religious background because of a long history of abuse based on group identity, and indeed discrimination on the basis of caste is outlawed. The very exercise of trying to determine what groups today have more of a claim to ancient heritage than others has not only contributed to conflict, but is also made almost meaningless in much of South Asia, due to the fact that the great majority of groups today are mixtures of the same populations whose ancestors have resided in the subcontinent for millennia^60,61^; however, there are cases where it is clear who is Indigenous, such as in the Andaman Islands^62^. There is a developed bureaucracy in many parts of South Asia for protecting cultural heritage, and working within this framework is an important mechanism for protecting communities from harm.

In West Eurasia, the suggestion that groups who claim local origins should have a special status has contributed to xenophobia and genocide. Nationalists promoting the idea of “blood and soil” in the Nazi period twisted archaeological research to legitimize land seizures by claiming that skeletons excavated in Eastern Europe had a “Germanic” morphology^63^. European archaeologists have worked for decades to deconstruct narratives that claim ownership of cultural heritage by specific groups. Ancient DNA ethics in a West Eurasian context must follow this movement away from the use of self-identified notions of ancestral connections to certain lands^63^, while simultaneously ensuring respect for the perspectives of national minorities that have been the subject of discrimination. The danger of government leaders citing archaeological and ancient DNA research to support favored narratives of group identity which can then be used to justify exclusionary policies is not just theoretical, but is an ongoing problem in some countries in West Eurasia today including in Hungary and Israel^65–67^.

## Five globally applicable guidelines for ethical ancient DNA research

We present five guidelines to promote robust ethical standards in ancient DNA research that apply across the breadth of research contexts discussed above, as well as other major world regions that we did not discuss due to space limitations including Central Asia, Siberia, East Asia, Southeast Asia, and Oceania (Box 1). We begin with guidelines that address issues of scientific ethics and then return to the topic of ensuring sensitivity of research to perspectives of communities, including Indigenous groups.

(1) Researchers must ensure that all regulations were followed in the places where they work and from which the human remains derived. Researchers must consider whether it is ethical to carry out ancient DNA research given the environment in the place from which they sample human remains. Once engaged in a project, researchers must abide by all local regulations. While this may seem obvious, the experience of some co-authors is that ancient DNA researchers have not always followed all agreements. For example, it may be necessary to obtain multiple levels of permission for scientific analysis or export of biological material from institutional, local, regional or national bodies, and to provide reports to curating institutions according to agreed timelines. Where local regulations are insufficient^68^, researchers must adhere to a higher standard following the principles below.

(2) Researchers must prepare a detailed plan prior to beginning any study. This should include an articulation of research questions; a description of the techniques to be used and expected impact on remains (including skeletal elements to be studied and quantity to be used); a description of the type of DNA data that will be generated; any plan for material sharing with collaborating laboratories; a timeline for the return of unused material and sharing of results; a plan for how, where, and by whom results will be disseminated; a plan for capacity-building or training in settings where this can be of value; and a plan for data storage and sharing agreed by stakeholders and complying with open data principles^69^. The plan should define the scope of the research and honestly communicate possible outcomes, recognizing that the analysis of genetic data can lead in unanticipated directions. Such a plan creates a record of the intended research that can be referred to later should there be a deviation from it. Adjustments to the study design should occur only with the support of those involved in the original agreement: researchers must acknowledge that when permission is granted to study the remains of ancient individuals, they become the stewards of that material for the purpose for which consent was obtained, but that ‘ownership’ is not transferred^70^. It is the responsibility of the researchers to share their plan with those responsible for the human remains and other groups whose perspectives need to be reflected; as such, it should be written in a way that is accessible to a non-specialist audience. If appropriate and agreed upon by all relevant parties, a pathway toward repatriation of human remains curated outside their area of origin may be outlined in the research plan.

(3) Researchers must minimize damage to human remains. Minimizing the impact of research on anthropological collections is especially important given the recent focus on a single skeletal element—the petrous bone—which often yields many-fold more human genetic data than other elements^71–74^. Researchers should develop a strategy through consultation with other stakeholders to balance concerns about protecting remains with their scientific analysis. Researchers should not collect human remains without training in best practice techniques to minimize damage while maximizing yield of useable data^11,12,75–78^. Researchers should not sample more material than necessary to be able to address their scientific questions, should provide documentation to those responsible for human remains noting when sampling occurred, and should report negative results to prevent repeated analysis using similar methods on remains with poor DNA preservation. Before sampling, morphology should be documented by high-resolution photography and bioarchaeological assessment. At least for very ancient individuals or those from unique contexts, micro-CT scans or casts should be produced, and there should be discussion about whether analysis of faunal or non-diagnostic remains should take place first to evaluate DNA preservation at a site.

Once sampling has occurred, responsible treatment of remains can also be promoted through the sharing of material as well as derived molecular products such as DNA extracts and libraries which reduces the need for additional sampling in subsequent studies. Researchers have the responsibility to maintain derived molecular products for the purposes of study replication. We also encourage researchers to seek approval for sharing sampled human remains and derived products between labs. This facilitates reappraisal of the questions addressed in the original study, as well as additional analyses beyond the scope of the initial study, as long as such uses are consistent with an approved research plan.

(4) Researchers must ensure that data are made available following publication to allow critical reexamination of scientific findings. Ancient DNA data must be published in a timely manner and subsequently made available at least for the purpose of critical reappraisal of results^79,80^. Scientists cannot ethically participate in a study if there is not a guarantee that data will be available at least for the purpose of verifying the accuracy of published findings, and this guarantee needs to be incorporated into the original permissions for the study. This is important both to prevent the spread of misinformation, and to enable future analyses that seek to re-examine the same questions.

It is best practice to make data fully available following publication, and indeed nearly all ancient genomic data have been published this way in enduring public data repositories, which has been an ethical strength of the field^81^. Beyond contributing to the advancement of scientific knowledge, making data fully available contributes to responsible stewardship of human remains, in that the ability to reuse data reduces the need for further sampling. However, we can envision scenarios in which discussions among stakeholders reveal that it would be ethical to limit the ways in which ancient DNA data can be reused, such as when reporting results from some types of analyses could harm stakeholders, which could outweigh the benefits of fully open data^6,10,82^. In these cases—which should be identified during a process of engagement prior to the inception of the study—the limitation of data distribution to qualified researchers who agree to only analyze the data for the purpose of reappraising the study findings should be part of the initial research plan.

When data are not made fully publicly available, management and distribution of data for the purpose of critical reexamination of results should be performed by an organization with expertise to prevent data misuse and without an interest in research outcomes. It has been suggested that stakeholders such as museums or Indigenous groups could be responsible for managing distribution of data after publication to researchers^10,13,15,83^. However, it is not consistent with professional ethics for researchers to participate in a study where those with a stake in the research findings can deny the sharing of data to qualified researchers whose goal is to critically re-examine the questions covered by the original research agreement. There are established mechanisms for ensuring distribution of non-fully public data to researchers who apply to use it for the purposes of critical re-examination. For example, data could be made available through a repository that shares data only upon formal application and approval from a data access committee that determines whether the applicant’s request satisfies the limitations on data use described in the publication, as is done for modern genomic data to address privacy concerns through mechanisms such as the dbGaP or EGA repositories^84,85^, although a shortcoming is that the data acquisition process can be slow^78^. Indigenous data bio-repositories are beginning to be established that involve communities in data storage and dissemination^10,13,81,86,87^, and while no stakeholder group—including researchers, communities representatives, or curators—should control the distribution of data to researchers who wish to critically re-examine questions covered in the original research agreement, Indigenous data bio-repositories could play an important role in storing and distributing data for purposes beyond those covered by the original research agreement.

(5) Researchers must engage with other stakeholders from the beginning of a study and ensure respect and sensitivity to stakeholder perspectives. A project to generate new ancient DNA data may be initiated by diverse stakeholders, including but not limited to local communities, archaeologists, anthropologists, geneticists, or curators, any or all of whom may be members of the research team if they contribute in a scholarly way to the work. Other stakeholders who are consulted should be thanked in the Acknowledgments sections of papers if they consent to be named. Stakeholders—ideally including groups from the place of origin of the human remains being studied—should be actively involved in discussions about study design, research questions, and whether a scientific project should proceed. Researchers must accept a negative answer if stakeholders are not collectively supportive of the work taking place^15^.

Once a consensus has been reached to proceed, professional scientific ethics requires that researchers are able to pursue their work up to the point of publication without requiring further approval. The suggestion that there should be a requirement for manuscripts to be approved by stakeholder groups who are not members of the research team prior to publication^15,83^ is not feasible, as researchers cannot ethically participate in a study in which this is mandated. The imperative of scientific independence once a study begins does not mean that researchers should publish results without considering stakeholder perspectives about the implications of the data. It is valuable to invite stakeholders to engage with research results through the addition of their perspectives or by providing critical feedback prior to publication especially when results are surprising and challenge previous assumptions. Continued engagement with other stakeholders after the beginning of a study is an effective mechanism by which researchers can address their professional ethical obligation to understand whether reporting a result in a particular way is likely to cause harm. If these conversations indicate that a result cannot be shared in a way that avoids significant harm to a stakeholder group, researchers should not publish such results.

Researchers should be available to provide regular updates and must commit to returning results at the culmination of a project. It should be made clear from the outset what the study’s potential findings may be, that genetic data may be inconsistent with other forms of knowledge, and that while the results of scientific analyses are reported as scholarly output, they do not discredit, diminish, or decrease the importance of traditional expertise and deeply held beliefs. Discrepancies between results from genetic analyses and other lines of evidence should be reported as important elements of the compound nature of understanding the past.

Researchers should commit to working with stakeholders on outreach efforts that create additional outputs accessible to communities. This may involve working with local collaborators to translate the results of papers into local languages^30,35,36,88,89^, developing children’s educational resources^90–93^, producing brochures and pamphlets for libraries or other community centers, or working with museums to design exhibits. When relevant, researchers should contribute to training and education, especially for members of stakeholder groups and local communities^4,15^, and should consider ways in which to improve the curatorial state of collections^11^.This can include supplying the resources needed for participating in the generation, interpretation, and dissemination of data, for example training in sampling of human remains or laboratory techniques, and financial support for further training or attending professional meetings. It is important for granting agencies to ensure that adequate funding is allocated to capacity-building initiatives.

## Promoting ethical DNA research on the remains of ancient individuals

As part of their work, scholars also have a broader obligation to correct ideologically-motivated distortions of research results. Following the technical presentation of data in academic publications, many studies are summarized by science journalists or educators for communication to broad audiences. There have been instances of journalistic and governmental misrepresentation of study findings for political ends, and scientists have an obligation to work to correct misinterpretation when appropriate^66^. Reaching out to the public can include writing essays and books, and contributing to social media and documentaries^94–102^.

Given the overwhelming support for these guidelines among the diverse participants in our workshop, we anticipate that the broader community engaged in ancient DNA research will be supportive of these principles as well, and suggest they could form a basis for official guidelines from journals, professional organizations, and granting agencies going forward.

## Box 1 Five Globally Applicable Guidelines for DNA Research on Human Remains

(1) Researchers must ensure that all regulations were followed in the places where they work and from which the human remains derived.
(2) Researchers must prepare a detailed plan prior to beginning any study.
(3) Researchers must minimize damage to human remains.
(4) Researchers must ensure that data are made available following publication to allow critical reexamination of scientific findings.
(5) Researchers must engage with stakeholders from the beginning of a study and ensure respect and sensitivity to other stakeholder perspectives.
